# Vitamin K-Dependent Carboxylation of Osteocalcin in Bone—Ally or Adversary of Bone Mineral Status in Rats with Experimental Chronic Kidney Disease?

**DOI:** 10.3390/nu14194082

**Published:** 2022-10-01

**Authors:** Marta Ziemińska, Dariusz Pawlak, Beata Sieklucka, Katarzyna Chilkiewicz, Krystyna Pawlak

**Affiliations:** 1 Department of Monitored Pharmacotherapy, Medical University of Bialystok, Mickiewicza 2C Str., 15-222 Bialystok, Poland; 2 Department of Pharmacodynamics, Medical University of Bialystok, Mickiewicza 2C Str., 15-222 Bialystok, Poland

**Keywords:** bone mineral status, chronic kidney disease (CKD), genes of vitamin VK cycle, vitamin K (VK), VK-dependent proteins, 5/6 nephrectomy model

## Abstract

Chronic kidney disease (CKD) commonly occurs with vitamin K (VK) deficiency and impaired bone mineralization. However, there are no data explaining the metabolism of endogenous VK and its role in bone mineralization in CKD. In this study, we measured serum levels of phylloquinone (VK1), menaquinone 4 and 7 (MK4, MK7), and VK-dependent proteins: osteocalcin, undercarboxylated osteocalcin (Glu-OC), and undercarboxylated matrix Gla protein (ucMGP). The carboxylated osteocalcin (Gla-OC), Glu-OC, and the expression of genes involved in VK cycle were determined in bone. The obtained results were juxtaposed with the bone mineral status of rats with CKD. The obtained results suggest that the reduced VK1 level observed in CKD rats may be caused by the accelerated conversion of VK1 to the form of menaquinones. The bone tissue possesses all enzymes, enabling the conversion of VK1 to menaquinones and VK recycling. However, in the course of CKD with hyperparathyroidism, the intensified osteoblastogenesis causes the generation of immature osteoblasts with impaired mineralization. The particular clinical significance seems to have a finding that serum osteocalcin and Glu-OC, commonly used biomarkers of VK deficiency, could be inappropriate in CKD conditions, whereas Gla-OC synthesized in bone appears to have an adverse impact on bone mineral status in this model.

## 1. Introduction

Chronic Kidney Disease Mineral and Bone Disorder (CKD-MBD) is a common complication of CKD, associated with abnormalities in bone metabolism and impaired bone mineralization [1,2,3]. In the course of CKD-MBD, both quality and quantity of bone tissue have been compromised, and the development of osteoporosis has an impact on a substantial increased risk of fractures and mortality in those patients [4,5].

Throughout life, bone undergoes a remodeling process in which the amount of resorbed bone should be equivalent to the amount of new bone formation and appropriate mineralization [6,7]. In the regulation of the mineralization process in CKD, many factors play a key role, such as parathormon (PTH), vitamin D, and vitamin K (VK) [8,9]. Vitamin K occurs in two forms—K1 (phylloquinone) and K2 (menaquinones, MKs). The most common MK in humans is the short-chain menaquinone 4 (MK4), which can be produced by endogenous conversion of phylloquinone to menaquinones with the enzyme UbiA prenyltransferase containing 1 (UBIAD1) [10,11,12]. The long chain MKs, such as menaquinone 7 (MK7), are found in fermented foods [13]. The pivotal role of vitamin K acts as a cofactor for the enzyme γ-glutamyl carboxylase (GGCX) in the gamma-carboxylation reaction, which in proper course is closely associated with its recycling, defined as the “vitamin K cycle”. The vitamin K epoxide is converted into the quinone form by vitamin K epoxide reductase complex subunit 1 (VKORC1). The transformation of the quinone to VK hydroquinone form occurs through quinone reductase. This form of VK can be reused as a cofactor for GGCX in the gamma-carboxylation reaction [14]. In the course of that reaction, carboxyl groups are added to Glu residues in proteins and transformed to Gla domains. This process transforms inactive (undercarboxylated) proteins into active (carboxylated) vitamin K-dependent proteins (cVKDPs), such as matrix Gla protein (MGP) and osteocalcin (OC) [15]. The increased circulating levels of undercarboxylated VKDPs—Glu-OC and ucMGP—reflect VK deficiency, and the measurement of these proteins is used to determine VK status [16,17].

Patients with CKD frequently suffer from subclinical VK deficiency, which may result from dietary restrictions, poor nutrient intake, and using drugs such as proton-pump inhibitors, steroids, statins, antihypertensives drugs, or broad-spectrum antibiotics, which decrease vitamin K synthesis by the impairment of the natural intestinal microflora [18,19,20,21,22]. Subclinical VK deficiency was confirmed in hemodialysis (HD) patients on the grounds of the high levels of ucOC, dephosphorylated-uncarboxylated MGP (dp-ucMGP), and low levels of VK1 [21,23]. In CKD, VK metabolism and recycling may also be impaired. McCabe et al. [24] assessed the genes expression of the VK cycle in a rat model of adenine-induced CKD and observed the reduced GGCX and VKORC1 expression in the thoracic aorta and a decreased level of UBIAD1 in the kidney.

Despite a small amount of research on the relationship between VK and bone health in CKD patients, it has been observed that low VK status has been linked to an increased risk of bone fracture [25,26] or reduced bone mineral density (BMD) [27,28,29,30]. Studies on the effect of VK supplementation on BMD are limited and inconclusive. In some research, an increase in BMD [31,32,33,34,35,36] or bone mineral content (BMC) [37] was observed under the influence of VK supplementation. However, in other studies, the above relationship was not shown [38,39]. MK7 supplementation reduced the levels of ucOC and dp-ucMGP in hemodialysis patients [40,41,42], whereas MK4 supplementation prevented bone loss in steroid-treated glomerulonephritis patients [43].

In the available literature, there is a lack of research explaining the role of endogenous VK in the bone mineralization process in the course of CKD. Therefore, the aim of the present study was the comprehensive assessment of endogenous VK metabolism in rats with CKD through determination of VK1, MK4, MK7, and undercarboxylated VKDPs—Glu-OC and ucMGP levels in serum, and the measurement of Gla-OC, Glu-OC levels, and their ratios in trabecular and cortical region of femurs. Then, the expression of genes related to VK recycling in bone were investigated. The obtained results were juxtaposed with the bone mineral status of uremic rats.

## 2. Materials and Methods

### 2.1. Animals

Twenty-six 4-week-old male Wistar rats were randomly assigned into two groups: the control group (CON, *n* = 10) subjected to sham-operation by renal decapsulation, and the experimental group (CKD, *n* = 16) after a two-step surgical 5/6 nephrectomy. During the experiment, animals were kept in conventional cages in vivarium conditions (humidity of 50%, 24 °C, and 12-h/12-h light/dark cycle) with unlimited access to sterilized tap water. The rats were fed a standard diet (Ssniff R/M-H) composed of 19% protein, 1% calcium, 0.70% phosphorus, 1000 IU of vitamin D3 per kg, and 5 mg of vitamin K as menadione per kg. The experiment’s overall time course lasted 24 weeks in order to monitor the development and progression of CKD. Finally, blood samples and femurs were obtained from the anesthetized animals and were secured for further studies. In the presented experiment, we employed materials from our previous research, where we gave a detailed description of animals’ characteristics, tissue collection, and applied procedures [44]. Briefly, markers of kidney function—serum urea and creatinine concentrations—were increased in CKD rats compared to CON (both *p* < 0.001). The animals with CKD had significantly higher levels of PTH (*p* < 0.01), whereas 1,25-dihydroxyvitamin D3 [1,25(OH)_2_D_3_] levels were comparable to healthy animals [44].

### 2.2. Measurement of Vitamin K Concentrations in Rat Serum

Vitamins K (i.e., phylloquinone (VK1), Menaquinone 4 (MK4), and Menaquinone 7 (MK7) were determined by the Laboratory of Perlan Technologies Polska, based in Gdynia, Poland. The following reagents and solvents from Merck KGaA were used: LC-MS grade water, LC-MS grade methanol, acetonitrile, hexane, bovine serum albumin (BSA), ammonium formate solution, formic acid, and standards of: vitamin K1 (phylloquinone), vitamins K2: MK4 (Menaquinone-4), and MK7 (Menaquinone-7) from Supelco Analytical Products(Merck Life Science Sp.z.o.o., Darmstadt, Germany), Deuterium-labelled internal standard of the vitamin K1-[d7] (phytonadione) were obtained from IsoSciences LLC (Ambler, PA, USA). Serum samples (100 μL) were spiked with Vitamin K1-[d7] ISTD, vortexed briefly, and acetonitrile was added to each tube, vortexed for 1 min, followed by hexane and again vortexed for 1 min. The upper organic layer was transferred to a new test tube and dried down under nitrogen at room temperature. The final dry extract was dissolved in acetonitrile and transferred to an MS vial. Aliquots of 10 μL were automatically injected into the HPLC system.

The Agilent Technologies 1260 LC system(Agilent Technologies, Santa Clara, CA, USA). was used for vitamins K analysis, including an autosampler, binary pump, and thermostated column compartment with G6470A Triple Quadrupole LC/MS (Agilent Technologies, Santa Clara, CA, USA). The sample separation was carried out on a reversed phase column Zorbax SB-C8 RRHT, 3.0 × 50 mm, 1.8 μm, 600 bar (Agilent Technologies, Santa Clara, CA, USA). The column temperature was kept constant at 30 °C. A gradient elution system was used with a flow rate of 0.4 mL/min. The binary gradient system consisted of 0.1% formic acid and 5 mM ammonium formate in water mobile phase (eluent A) and methanol acidified with 0.1% formic acid (eluent B). Gradient elution was applied as follows: 0 min—70% A, 30% B; 2 min—10% A, 90% B; 3 min—0% A, 100% B; 15 min—0% A, 100% B; 20 min—70% A, 30% B. The AJS ESI ion source operated in positive ion mode with drying gas temperature of 330 °C and 8 L/min gas flow. The sheath gas temperature was set to 320 °C with a flow of 11 L/min. Nitrogen was used as a nebulizer gas (45 psi) and ultrahigh-purity nitrogen was used as collision gas. The capillary and nozzle voltages were 3000 V and 1000 V, respectively. Identification and quantification were based on MS/MS multiple reaction monitoring (MRM). An overview of the MRM transitions, collision energies, and retention time for the analytes is given in Table 1. All aspects of system operation and data acquisition were controlled using Agilent MassHunter Workstation Software (version B.10.00).

### 2.3. Serum and Bone Levels of Vitamin K-Dependent Proteins

Undercarboxylated osteocalcin (Glu-OC), carboxylated osteocalcin (Gla-OC), and total osteocalcin were determined by ELISA kits: Rat Glu-Osteocalcin High Sensitive EIA, Rat Gla-Osteocalcin High Sensitive EIA (Takara Bio Inc., Shiga, Japan), and Rat Osteocalcin ELISA kit from Immutopics, Inc., San Clemente, CA, USA, respectively. Rat undercarboxylated Matrix Gla Protein (ucMGP) was quantified in serum by ELISA kit from MyBioSource, Inc., San Diego, CA, USA, according to the manufacturer’s recommendations. Trabecular and cortical concentrations of Gla-OC and Glu-OC were adjusted for protein concentration, and the ratio Gla-OC/Glu-OC was calculated.

### 2.4. Preparation of Bone Tissue Homogenates

The procedure of the preparation of 10% homogenates from bone tissue, using a high-performance homogenizer (Ultra-Turrax T25; IKA, Staufen, Germany) with a stainless-steel dispersing element (S25N-8G; IKA), has been described in detail previously [45]. Briefly, segments of bone tissue were taken from the femoral diaphysis and distal femoral epiphysis (from the cortical bone region and trabecular bone region, respectively). Collected materials were weighed, closely rinsed, and then homogenized in the cold potassium phosphate buffer (50 mM, pH = 7.4; POCh). The supernatant was obtained by the centrifugation of 10% homogenates for 10 min at 700× *g* at 4 °C and stored at −80 °C.

### 2.5. Quantitative Real-Time Polymerase Chain Reaction (RT-PCR)

Total RNA was isolated from femoral tissue using Thermo Scientific GeneJET RNA Purification Kit (Thermo Scientific, Vilnius, Lithuania), and Quantitative RT-PCR was performed as previously described [46]. Briefly, using the Thermo Scientific NanoDrop 2000 spectrophotometer (Waltham, MA, USA), RNA was quantified, and its quality was confirmed. The RevertAid™ First Stand cDNA Synthesis Kit (Thermo Fisher Scientific, Waltham, MA, USA) was used to reverse-transcribe total RNA (1 μg). In order to perform quantitative real-time PCR, the SYBR Green Master Mix was used (EURx, Gdańsk, Poland), and relative quantification of gene expression was performed by the comparative CT method (ΔΔCT). Glyceraldehyde 3-phosphate dehydrogenase (GAPDH) was employed as a housekeeping gene to normalize expression level [46]. Primers were designed using Primer-BLAST software(Primer3 program). The primer sequences were (5′-3′ forward-reverse): VKORC1 (5′-TCCCGCGTCTTCTCCTCTC-3′; 5′-CCAACGTCCCCTCAAGCAAC-3′), GGCX (5′-GGATGCTGACTGGGTTGAGG-3′; 5′-GCTCCTCCGACAACACTAGC-3′) and UBIAD1 (5′-GCTGTGTGTGTGCTGCTTAC-5′; CCCAGTGCCACGTACTTGAA-3′).

### 2.6. Genes of Osteoblastogenesis

The expression of key genes involved in osteoblastogenesis, such as: Forkhead Box Transcription Factor 1 (FOXO1), Activating Transcription Factor 4 (ATF4), Runt-Related Transcription Factor 2 (RUNX2), Alkaline Phosphatase (ALP), and Bone Gamma-Carboxyglutamate Protein (BGLAP) were determined previously [46].

### 2.7. The Mineral Status of Femurs

Using the appropriate small animal software, bone densitometry scans were carried out on a Horizon QDR Series X-ray Bone Densitometer (Hologic Inc., Bedford, MA, USA). For all rats were conducted whole-femur measurements—bone mineral area, bone mineral density (BMD), and bone mineral content (BMC). Moreover, at the distal metaphysis (R1 region) and midshaft (R2 region), the subregional BMC and BMD of small uniform areas were quantified. The R1 region is constituted of mixed trabecular and cortical bone, whereas the R2 region represents cortical bone. The results of these densitometric measurements have been shown elsewhere [44].

### 2.8. Statistical Analysis

Shapiro–Wilk tests were performed to determine if continuous variables were normally distributed. Normally distributed data were expressed as mean ± SD. Non-Gaussian data were presented as median (25th to 75th percentiles). The two groups (CON and CKD) were compared by using an unpaired *t*-test with Welch correction or nonparametric Mann–Whitney Test. *p* < 0.05 was the accepted level of significance. The correlations between study variables were calculated using Spearman’s rank correlation analysis. Computations were performed using Statistica ver.13 software (StatSoft, Tulsa, OK, USA), and the graphic design presentation of the results was performed using GraphPad Prism 6.0 software (San Diego, USA).

## 3. Results

### 3.1. The Status of Endogenous Vitamin K in Rats with CKD

**Figure 1:**
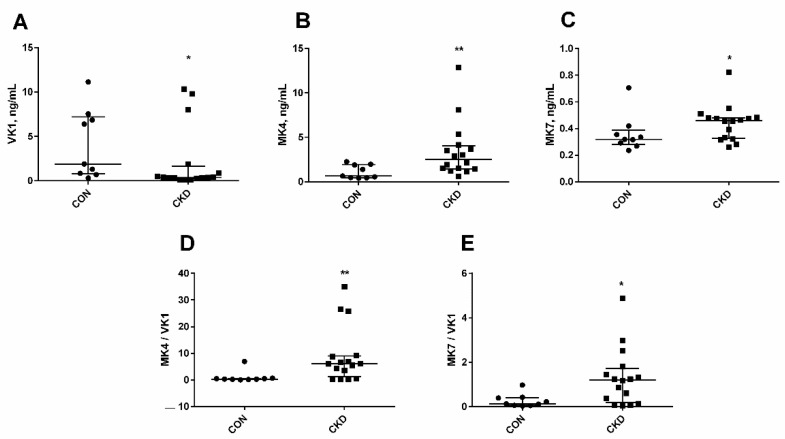
Vitamin K1 (**A**), MK4 (**B**), MK7 (**C**) concentrations, and the ratios of MK4/VK1 (**D**) and MK7/VK1 (**E**) in serum rats with chronic kidney disease (CKD) and healthy controls (CON) fed with a standard rodent diet. The lines correspond to the 25th and 75th percentiles and the median. * *p* < 0.05, ** *p* < 0.01 CON versus CKD rats; VK1—phylloquinone; MK4—Menaquinone 4; MK7—Menaquinone 7; the circles represent results in controls; the squares represent results in CKD.

As has been shown in Figure 1, the levels of vitamin K were changed in chronic kidney disease. The concentration of VK1 in CKD was lower than in controls (Figure 1A). In contrast, we observed a considerable increase in MK7 (Figure 1B), especially in MK4 levels (Figure 1C) in uremic rats. The ratio of MK7/VK1 and MK4/VK1 indicates how efficiently phylloquinone is converted to menaquinone (VK2). In the present study, a significant rise in the above ratios was shown, particularly in MK4/VK1 ratio in CKD compared with the controls (Figure 1D,E).

### 3.2. Serum Levels of Vitamin K-Dependent Proteins in Rats with CKD, and the Impact of Kidney Function and PTH on Their Concentrations

**Figure 2:**
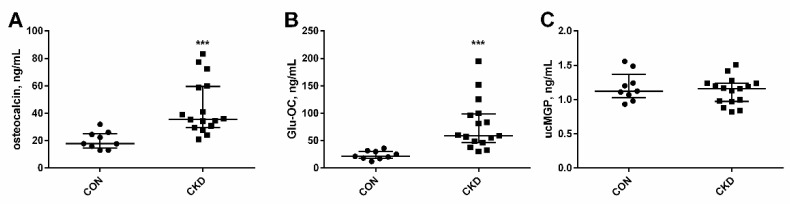
Serum levels of vitamin K-dependent proteins: osteocalcin (**A**), Glu-OC (**B**) and ucMGP (**C**) in rats with chronic kidney disease (CKD) and healthy controls (CON). The lines correspond to the 25th and 75th percentiles and the median. *** *p* < 0.001 CON versus CKD rats; Glu-OC—undercarboxylated osteocalcin; ucMGP—undercarboxylated Matrix Gla Protein.

The concentrations of total osteocalcin, its undercarboxylated form (Glu-OC), and uncarboxylated matrix Gla protein (ucMGP) are shown in Figure 2. These proteins reflect VK status and are used as markers of VK deficiency. A significant increase was observed in both total OC and Glu-OC in rats with CKD in comparison with healthy animals (Figure 2A,B). However, there was no difference in the ucMGP concentration between uremic rats and controls (Figure 2C).

**Figure 3:**
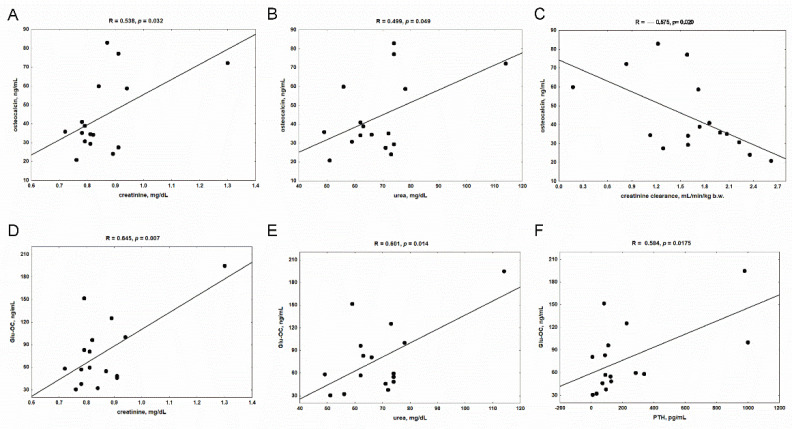
The associations between serum levels of osteocalcin and its undercarboxylated form (Glu-OC), and markers of kidney function: creatinine (**A**,**D**), urea (**B**,**E**), creatinine clearance (**C**) and parathyroid hormone (PTH) concentration (**F**) in rats with chronic kidney disease (CKD).

Both total osteocalcin and its undercarboxylated form (Glu-OC) in serum were positively correlated with kidney function markers: creatinine and urea concentrations in CKD rats (Figure 3A,B,D,E). The inverse relationship was observed between total osteocalcin and creatinine clearance (Figure 3C), whereas circulating Glu-OC was related to PTH concentrations (Figure 3F).

### 3.3. The Levels of Glu-OC, Gla-OC, and Gla-OC/Glu-OC Ratios in Femoral Bone Tissue of Rats with CKD, and Their Relations with Serum Glu-OC

**Figure 4:**
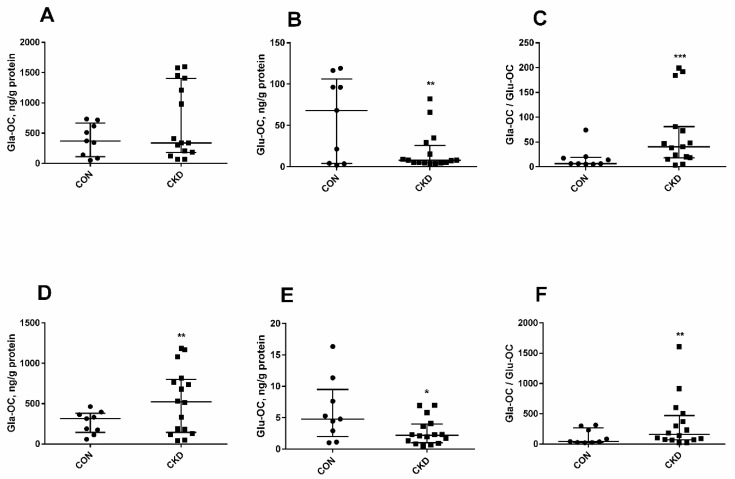
The levels of carboxylated osteocalcin (Gla-OC), undercarboxylated osteocalcin (Glu-OC) and their ratios (Gla-OC/Glu-OC) in trabecular (**A**–**C**) and cortical (**D**–**F**) femoral bone tissue. The lines correspond to the 25th and 75th percentiles and the median. * *p* < 0.05, ** *p* < 0.01, *** *p* < 0.001 CON versus CKD rats; CKD—chronic kidney disease; CON—controls.

In the current study, Gla-OC and Glu-OC concentrations and the Gla-OC/Glu-OC ratio were measured in trabecular (Figure 4A–C) and cortical (Figure 4D–F) bone tissue. Gla-OC levels in trabecular bone did not differ between the studied groups (Figure 4A), whereas Glu-OC level was considerably lower in CKD than in controls (Figure 4B). It is interesting to note that in CKD, the Gla-OC level in cortical bone tissue significantly increased (Figure 4D), while the Glu-OC concentration was reduced when compared to healthy animals (Figure 4E). Moreover, in both bone areas, the Gla-OC/Glu-OC ratios in uremic rats were considerably higher than in CON (Figure 4C,F).

**Figure 5:**
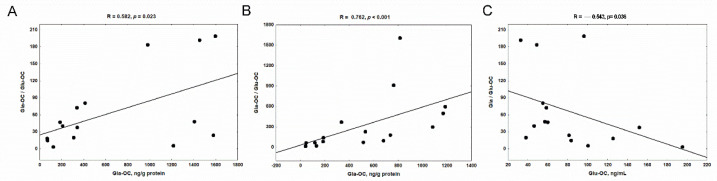
The associations between carboxylated osteocalcin (Gla-OC) and Gla-OC/Glu-OC ratio in trabecular (**A**) and cortical (**B**) bone tissue; the relations between trabecular Gla-OC/Glu-OC ratio and serum undercarboxylated osteocalcin (Glu-OC) levels; (**C**) in rats with chronic kidney disease (CKD).

In CKD group, we observed a positive association between Gla-OC concentrations and Glu-OC/Gla-OC ratios in trabecular (Figure 5A) and in cortical (Figure 5B) bone regions. The weak correlation was also noticed between Glu-OC and Gla-OC levels in trabecular (R = 0.512, *p* = 0.045), but not in cortical bone tissue (R = 0.105, NS). Among the analyzed parameters, only the Gla-OC/Glu-OC ratio in the trabecular bone region was inversely correlated with the concentration of Glu-OC in the serum (Figure 5C).

### 3.4. The Expression of Genes Coding Vitamin K Cycle Enzymes in Femurs of Rats with CKD, and Their Associations with Genes Participating in Osteoblastogenesis

**Figure 6:**
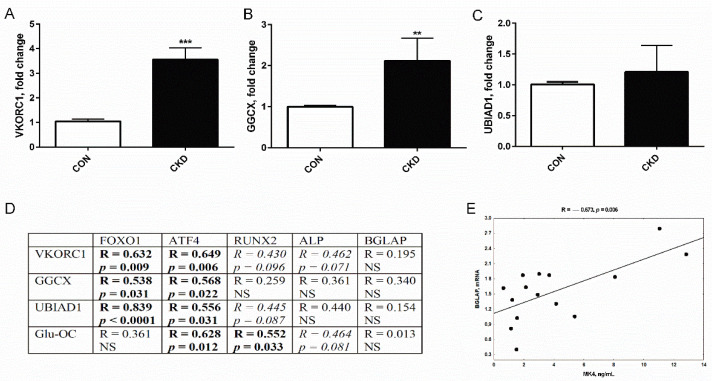
The expression of genes of vitamin K cycle enzymes in femurs of rats with chronic kidney disease (CKD) (**A**–**C**), and their associations with the expression of genes of osteoblastogenesis and trabecular Glu-OC levels (**D**), as well as the relationship between serum MK4 level and BGLAP expression in bone of rats with CKD (**E**). ** *p* < 0.01, *** *p* < 0.001 CON versus CKD rats; CON—controls; VKORC1—vitamin K epoxide reductase complex subunit 1; GGCX—the gamma-glutamyl carboxylase; UBIAD1—UbiA prenyltransferase domain-containing protein 1; FOXO1—Forkhead box transcription factor 1; ATF4—activating transcription factor 4; RUNX2—Runt-related transcription factor 2; ALP—alkaline phosphatase; BGLAP—bone gamma-carboxyglutamate protein; Glu-OC—undercarboxylated osteocalcin; MK4—menaquinone 4; the bold text indicates statistically significant correlations; italic text indicates tendency to correlation.

The expression of VKORC1 and GGCX gene was significantly higher in uremic rats compared to the control group (Figure 6A,B), whereas the increase in the UBIAD1 gene did not achieve a statistically significant level (Figure 6C).

Our previous research performed on the bones of rats with CKD established that the expression of FOXO1, ATF4, RUNX2, and ALP, which are the key genes responsible for the initial phases of osteoblast development, was significantly increased, whereas the expression of BGLAP, which is linked to the late stage of osteoblast differentiation, was only slightly elevated [46]. As has been shown in Figure 6D, the expression of the genes involving in VK cycle (especially VKORC1 and UBIAD1) was strongly and positively correlated with the expression of the genes of the early stages of osteoblast development. The tendency of these positive relationships was also seen between VK cycle genes expression and the markers of osteoblast differentiation, such as RUNX2 and ALP. Interestingly, we also observed the association between trabecular Glu-OC levels and the expression of genes reflecting the osteoblast differentiation (ATF4, RUNX2, ALP). Other interesting observations were the lack of the correlation between BGLAP expression and the genes belonging to VK cycle and trabecular Glu-OC levels (Figure 6D), as well as the strong positive association between BGLAP mRNA and circulating MK4 level (Figure 6E).

### 3.5. The Relationship between the Expression of VKORC1 and the Concentration of Gla-OC in Trabecular Bone Tissue and Serum Glu-OC

**Figure 7:**
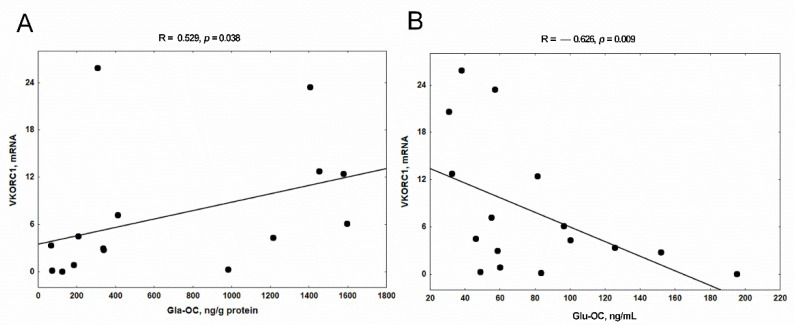
The association between bone VKORC1 expression and Gla-OC levels in trabecular bone (**A**), and VKORC1 expression and Glu-OC levels in serum of rats with CKD (**B**). MK4—menaquinone 4; BGLAP—bone gamma-carboxyglutamate protein; VKORC1—vitamin K epoxide reductase complex subunit 1; Gla-OC—carboxylated osteocalcin; Glu-OC—undercarboxylated osteocalcin; CKD—chronic kidney disease.

Next, we analyzed the relations between the expression of VK cycle enzymes and both forms of osteocalcin in femoral bone of rats with CKD. There was no correlation between GGCX and the levels of Gla-OC and Glu-OC. Surprisingly, we found a positive relationship between VKORC1 expression and Gla-OC concentration in trabecular bone tissue (Figure 7A), as well as a strong inverse relationship between VKORC1 expression and serum Glu-OC concentration (Figure 7B).

### 3.6. The Associations between Bone Glu-OC, Gla-OC, and Bone Mineral Status in Rats with CKD

Rats with CKD had significantly decreased densitometric parameters compared with controls in the whole-femur measurements, namely in the bone mineral area (*p* < 0.05), BMC, and BMD (both *p* < 0.01). These differences were particularly seen in the metaphyseal area (R1) of femurs, where the values of BMC and BMD were significantly decreased in rats with CKD compared to controls (both *p* < 0.001). In contrast, mineral status measured in the diaphyseal area (R2) was only slightly impaired—the values of BMC were lower in CKD than in controls (*p* < 0.01), whereas BMD did not differ between the studied groups (for details please see our previous study [44]).

**Figure 8:**
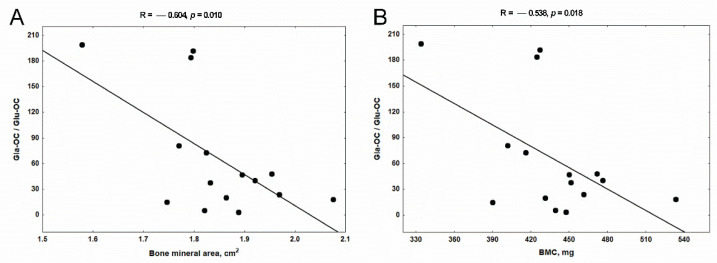
The associations between Gla-OC/Glu-OC ratio in trabecular bone tissue and the parameters of mineral status: bone mineral area (**A**) and BMC (**B**) of femoral bone of rats with chronic kidney disease (CKD). Gla-OC—carboxylated osteocalcin; Glu-OC—undercarboxylated osteocalcin; BMC—bone mineral content.

In the present study, we analyzed the associations between both forms of osteocalcin and their ratios measured in trabecular and cortical bone tissue, with these densitometric parameters of femurs. At the level of the trabecular bone, we have demonstrated that only Gla-OC/Glu-OC ratio was strongly and inversely related to bone mineral area (Figure 8A) and BMC (Figure 8B) in uremic rats.

**Figure 9:**
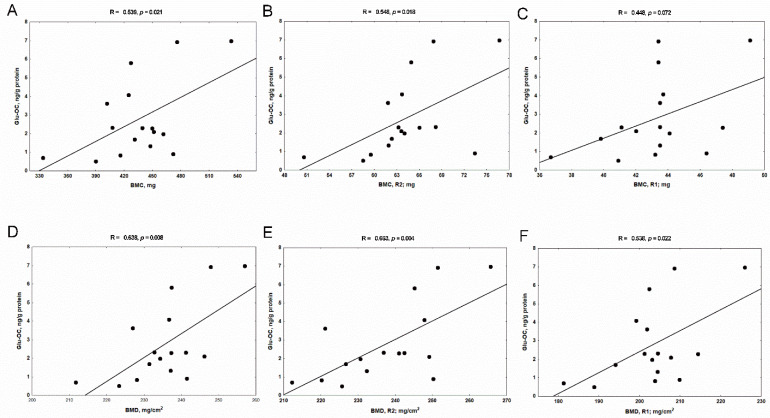
The associations between undercarboxylated osteocalcin (Glu-OC) levels in cortical bone tissue and whole BMC, BMD (**A**,**D**), diaphyseal BMC, BMD (**B**,**E**) and metaphyseal BMC, BMD (**C**,**F**) of femurs of rats with chronic kidney disease (CKD). BMC—bone mineral content; BMD—bone mineral density; R1—metaphyseal area; R2—diaphyseal area of femur.

Surprisingly, at the level of the cortical bone, we observed that only Glu-OC values were positively associated with the whole-femur values of BMC (Figure 9A) and BMD (Figure 9D). These relations were particularly strong in the diaphyseal area (R2), which is rich in cortical bone (Figure 9B,E), but they also were noticeable in the metaphyseal area (R1) of femurs (Figure 9C,F).

## 4. Discussion

Although a high prevalence of subclinical vitamin K (VK) deficiency has been described in patients with CKD [18,19,20,21,22] and there are suggestions that this condition can impact bone quality [47,48], there are a lack of experimental data on this issue. To the best of our knowledge, this is the first comprehensive study in which we determined the endogenous VK metabolism, the bone levels of VK-dependent proteins (VKDPs), and the expression of VK cycle enzymes in femoral bone of rats with 5/6 nephrectomy. For determination of the impact of endogenous vitamins K on bone mineralization in this CKD model, the obtained results were juxtaposed with the parameters of the mineral status of femurs.

Among the K vitamins, the concentrations of phylloquinone (VK1), menaquinone 4 (MK4), and menaquinone 7 (MK7) were measured in rats’ serum. Despite the fact that all rats have received the same standard diet supplemented with menadione, the VK status of uremic rats was altered, with a decrease in VK1 concentration and a significant increase in MK7, particularly in MK4 levels. VK1 is the major dietary form of VK, and the primary form found in circulation [49]. VK1 is endogenously converted to MK4 via a menadione intermediary [50,51,52]. Hirota et al. [52] were the first to show that the release of menadione from VK1 and its conversion to MK4 occurred in the intestine of rats, therefrom MK4 entered the blood circulation through the mesenteric lymphatic system. On the other hand, this same group proposed that menadione from intestine is delivered via the mesenteric lymphatics into the blood and then transported to peripheral tissues, where it is transformed into MK4 by UBIAD1. Details about MK7 distribution and metabolism in rats are still limited. Ikeda et al. [53] proposed that MK7 can also be a precursor of MK4 and may be converted to MK4 in the body. Regardless of how MK4 comes to be formed, it is believed that rodents consuming commercially available rodent chow, fortified with menadione (a precursor for MK4), have sufficient vitamin K for all tissue functions [54].

VK1 deficiency has been observed in CKD patients and animals with experimental CKD [24,48,55], and in this respect our results are in line with these earlier observations. However, the ratios of MK7/VK1 and MK4/VK1, which provide an index of the efficiency of conversion of VK1 to vitamins K2 [56], were increased in uremic animals compared to controls. This suggests that lower VK1 levels observed in CKD rats could be a result of greater tissue utilization of this vitamin, possibly reflecting an increased physiological demand, especially for MK4, during uremia. The similar phenomenon was previously described by Ferland et al. [57] in rats fed different VK1 diets during aging.

The measurement of serum levels of VKDPs, like total osteocalcin and its undercarboxylated form, Glu-OC, are sensitive indirect tests in detecting subclinical VK deficiency [16]. Both total osteocalcin and Glu-OC were markedly increased in the serum of CKD rats in this experiment, and similar findings have been consistently observed by others in human and experimental CKD [19,24,48]. It is generally believed that the high percentage of Glu-OC reflects insufficient VK intake, low VK status, and impaired bone mineralization [28,29,30]. In the present study, we observed the direct associations between kidney function markers and total osteocalcin, indicating that this parameter can be accumulated in the blood of CKD animals independently of its release from bone. This observation is in accordance with the study of Price et al. [58], who demonstrated that nephrectomy can block the serum clearance of osteocalcin. In turn, renal insufficiency combined with an increase in PTH levels seems to affect serum Glu-OC concentrations in our rats with CKD. It is possible that PTH may enhance the synthesis of Glu-OC by the stimulation of osteoblastogenesis, as has been reported previously [59,60,61,62]. Thus, the increased serum Glu-OC in the condition of uremia and hyperparathyroidism, observed in this study, does not necessarily reflect VK deficiency. A good alternative to determine VK status is measurement serum ucMGP, which is a recognized marker of VK status in nephrectomized rats [63]. In contrast to Glu-OC, ucMGP concentration did not differ between the studied groups in this study. All these data indicate that VK status was sufficient in CKD animals.

Osteocalcin is synthesized in osteoblasts, and its three-glutamate residues (Glu-OC) are posttranslationally converted into γ-carboxyglutamate (Gla-OC) by VK, which is a cofactor of GGCX [64,65]. Gla-OC strongly binds to hydroxyapatite in bone, whereas Glu-OC, which does not have this ability, is released into the blood [58]. We measured the Gla-OC, Glu-OC, and Gla-OC/Glu-OC ratio, being an index of the availability of VK at the bone level, in trabecular (Figure 4A–C) and cortical (Figure 4D–F) bone tissue. In both bone regions the Gla-OC/Glu-OC ratios were increased, and Glu-OC concentrations were reduced in CKD rats compared to controls. These results indicate the accelerated Glu-OC to Gla-OC transformation in bone of CKD rats, which increased Gla-OC while consuming Glu-OC [66], reflecting sufficient or even elevated levels of VK in their bones. However, in trabecular bone of uremic rats, Gla-OC level was comparable with that of controls, and the only weak relationship existed between Gla-OC and Gla-OC/Glu-OC ratio. In contrast, the Gla-OC level rose significantly, and it was strongly correlated with Gla-OC/Glu-OC ratio in the cortical bone region of these animals. These data support our new observation: that the generation of Gla-OC in trabecular bone, in which a more rapid bone turnover rate occurs, was not as effective as in cortical bone. Interestingly, serum Glu-OC levels were inversely related to trabecular Gla-OC/Glu-OC ratio, indicating that the Gla-OC generation process in trabecular bone region is an important factor influencing the serum Glu-OC level.

Vitamin K undergoes a cycle of oxidation followed by reduction through the “vitamin K cycle”, which includes VKORC1, GGCX, and UBIAD1 enzymes [65,67]. During γ-carboxylation of Glu-OC, VK is converted into VK epoxide by GGCX [65], and VK epoxide can then be reduced by the enzyme VKORC1 to VK-hydroquinone, which can be used once again for the carboxylation reaction [65,67]. The UBIAD1 gene encodes an MK4 biosynthetic enzyme, which is responsible for VK1 to MK4 bioconversion in extrahepatic tissues [68].

The expressions of VK cycle enzymes were previously determined in different tissues of rats with adenine-induced CKD, and in general, the decreased recycling and utilization of VK was found [24,69,70]. To the best of our knowledge, this is the first study that measured the expression of genes involved in VK recycling at the bone level of uremic rats. There was a significant increase in the expression of VKORC1 and GGCX in the femurs of CKD rats compared to controls, whereas there was no difference in the expression of UBIAD1. These results indicated that VK1 to MK4 bioconversion, as well as MK4 recycling and utilization, can occur in the bones of CKD rats even more effectively than in healthy animals. Surprisingly, regarding Gla-OC levels in the studied bone regions, it seems that the enzymes of VK cycle should act better in a cortical, less metabolically active part of the bone than in the trabecular, which is characterized by more active bone turnover.

Previously, we observed the alterations in osteoblastogenesis process in bone of our rats with CKD, with the significantly increased expression of genes involved in the early stages of osteoblastogenesis, like FOXO1, ATF4, RUNX2, and ALP, whereas the expression of marker of the late stage of osteoblast differentiation, BGLAP, was only slightly increased. This phenomenon resulted in impaired osteoblast maturation and decreased bone mineral status [46]. Our findings were in line with clinical observation of Pereira et al. [71], who also showed that the increase of osteoblastic markers occurred simultaneously with low osteocytic gene expression in cells obtained from dialyzed patients. In the present study, we analyzed the associations between the expression of VK cycle enzymes and mentioned the above genes involved in the individual stages of osteoblastogenesis. As has been presented in Figure 6D, the expression of enzymes of VK cycles occurred in the early stages of osteoblastogenesis. What is more, Glu-OC levels in trabecular bone tissue were also associated with the early markers of osteoblast proliferation/differentiation, but not with BGLAP. Consistent with previous reports, PTH may accelerate differentiation of osteoprogenitor cells into osteoblasts [72]. Because PTH level was increased in serum of our CKD rats [44], and previously we showed that endogenous PTH may influence the expression of genes engaged in the early stage of osteoblastogenesis in young uremic rats [73], we speculate that this hormone could also contribute to the increased expression of VK cycle enzymes in this study. However, BGLAP expression increases significantly at the late stage of osteoblast differentiation [74], and this fact could explain the lack of association between BGLAP and the expression of VK cycle enzymes and bone Glu-OC generation. Interestingly, we noticed that circulating MK4 was positively associated with BGLAP expression in bone (Figure 6E). This is in accordance with the findings of Weng et al. [66], who evaluated the effect of MK4 on osteocalcin expression in calvarial bone defect repair in osteopenic rats. These and the above-presented results (Figure 6) suggest that in CKD conditions, the VK recycling, expression of BGLAP, and generation of different forms of osteocalcin may occur in the distinct stages of osteoblastogenesis under the control of endogenous PTH and MK4. Thus, the lack of difference in trabecular Gla-OC levels observed in the present study could be a result of the individual effects of PTH, which stimulates the synthesis of osteocalcin [60]; and MK4, which increased Gla-OC while consuming Glu-OC. In agreement with this hypothesis, the combined MK4 and PTH_1-34_ treatment increased serum Gla-OC/Glu-OC ratio in osteopenic rats, however the highest Gla-OC/Glu-OC ratio was observed not in the combined group, but in the MK4-treated group [66].

Another interesting finding of the present study is the association between VKORC1 mRNA levels and Gla-OC concentration in the trabecular bone region of uremic rats. In contrast, such relations were not noticed between GGCX and both forms of osteocalcin in the bone regions analyzed by us. The posttranslational modification of Glu-OC to Gla-OC inside osteoblasts generally involves two enzymes, GGCX and VKORC1, which together constitute the VK cycle. GGCX requires a reduced form of VK as an obligate cofactor, and γ-carboxylation is dependent on the reduction of VK epoxide by a VKORC1 [65]. Ferron et al. [75], using cell-specific gene inactivation models, demonstrated that VKORC1 is required for γ-carboxylation of osteocalcin in osteoblasts. They also showed that animals lacking GGCX or VKORC1 presented almost no osteocalcin in their bones, providing the first in vivo evidence that the generation of Gla-OC is necessary for its accumulation in the mineral component of bone. This is consistent with the inverse relationship between bone VKORC1 gene expression and serum Glu-OC levels observed in our uremic rats (Figure 7B), confirming the significance of VKORC1 in the process of Gla-OC formation in conditions of CKD.

The second aim of the present study was to establish the potential consequence of VK-dependent mechanisms for the bone mineral status of uremic rats. Previously, we showed that CKD development resulted in significantly decreased densitometric parameters in our uremic animals compared with controls in whole-femur measurements, particularly in the metaphyseal area (R1). In contrast, mineral status measured in the diaphyseal area (R2) was damaged to a lesser extent [44]. In the present study, we juxtaposed the parameters of bone mineral status obtained in this previous study [44] with the concentrations of VK-dependent proteins: Gla-OC and Glu-OC in the corresponding regions of the femur. Unexpectedly, the Gla-OC/Glu-OC ratio in trabecular bone tissue was inversely associated with the mineral status of femurs (Figure 8). In turn, the positive relations were observed only between Glu-OC levels in cortical bone tissue and the parameters of bone mineral status, which were strongest in the diaphyseal area (R2) of femurs (Figure 9). Although Gla-OC, due to its specific interaction with hydroxyapatite, is thought to be associated with bone mineralization [76,77,78], there is also evidence showing that Gla-OC is not critical for bone mineralization or even that it functions as a “negative regulator of skeleton” [79]. Genetic osteocalcin depletion does not change the mineral content of bone matrix in mice [80]. Complete loss of osteocalcin resulted in bones with significantly increased trabecular thickness, density, and volume, whereas cortical bone volume and density were not increased in osteocalcin-null male rats [79]. The treatment with warfarin, despite significantly lowering Gla-OC levels, did not reduce the mineral content of fracture calluses [59], and is not directly linked with BMD of rat bone [81]. Amizuka et al. [82] demonstrated that osteocalcin is not related to mineral deposition but does participate in the growth and maturation of hydroxyapatite. A recent study by Simon et al. [83] demonstrates that osteocalcin takes on the functions of Ca-ion transport and suppression of hydroxyapatite expansion. An interesting study by Uchida et al. [84] revealed that the commensal microbiota prevents excessive bone mineralization by stimulating osteocalcin expression in osteoblasts, enhancing both osteoblast and osteoclast activity.

Assuming that Gla-OC is the marker of mature osteoblast [84], the relations observed in this study between bone mineral status and Glu-OC to Gla-OC transformation are compatible with the results of these teams, which demonstrated that Gla-OC may act as an inhibitor of bone mineralization [79,80,81,82,83,84]. We believe that in the trabecular bone region, PTH-dependent acceleration of osteoblastogenesis resulted in the generation of immature osteoblasts with insufficient Gla-OC production, which may lead to decreased bone mineral status [46]. In cortical bone, where Gla-OC level was approximately 1.5 times that in trabecular bone, the osteoblasts should be more mature. However, the mineral status in this bone region was directly associated with Glu-OC, representing the part of osteocalcin, which was not transformed to Gla-OC. The above results indicate an unfavorable role of Gla-OC in the mineralization of long bones in CKD conditions.

Some limitations should be considered in the interpretation our results. Firstly, the cross-sectional design of this study does not determine whether a causal relationship exists between VK metabolism in bone and the parameters of bone mineral status. Secondly, we cannot exclude the possibility that the observed associations could be partially attributed to other factors not considered in this study that may affect osteoblast proliferation/differentiation and bone mineral status, such as the metabolites of tryptophan [44,85]. Thirdly, gonadectomy was not performed in this study, so contributions to mineral and bone metabolism of sex hormones, especially androgen in the rats, were not examined.

A major strength of our study is the measurement of circulating vitamins K together with the expression of VK recycling enzyme in bone, and Glu-OC, Gla-OC directly in the appropriate bone tissue, as the determination of these compounds in blood may not accurately represent the bone microenvironment [81]. Moreover, the studied groups were homogeneous with regard to age, sex, diet, and the absence of medication.

## 5. Conclusions

This study presents a comprehensive assessment of the metabolism of endogenous vitamin K, VK recycling in bone, VKDPs at serum and bone levels, and their impact on bone mineral status of rats with CKD. The obtained results indicate that the reduced level of VK1 observed in rats with CKD may be caused by the accelerated conversion of this vitamin to the form of menaquinones. The measurement of serum osteocalcin and Glu-OC, commonly used as the indicators of VK deficiency, seems to be ineffective in CKD conditions, especially in the presence of hyperparathyroidism. For the first time, we showed that bone tissue possesses a set of enzymes that allows for the conversion of VK1 to the form of menaquinone, as well as to recycling of VK. However, in the course of CKD with hyperparathyroidism, despite the appropriate conditions for the formation of active forms of VK, the intensified process of osteoblastogenesis causes the generation of immature osteoblasts with impaired mineralization capacity. Of particular clinical significance seems to be the fact that Gla-OC formed at the bone level turned out to be inversely correlated with bone mineral status in this model. This sheds new light on the metabolism of endogenous VK and its importance in the process of bone mineralization in CKD, particularly in patients with hyperparathyroidism.

## Figures and Tables

**Table 1:** LC-MS/MS parameters.

	Precursor ion (*m*/*z*)	Production (*m*/*z*)	Collision Energy (V)	Retention Time (min)
K1	451.4	187.2	26	6.02
K1-d7	458.4	194.3	26	6
MK4	445.3	187.3	18	5.42
MK7	649.5	187.2	38	7.35

K1*—*phylloquinone; K1-d7*—*deuterium-labelled internal standard of the vitamin K1; MK4*—*Menaquinone-4; MK7*—*Menaquinone-7.

## Data Availability

Data is stored by corresponding author and may be share upon request.

## Abbreviations

1,25 (OH)_2_D	1,2-dihydroxyvitamin D
ALP	alkaline phosphatase
ATF4	activating transcription factor 4
BGLAP	bone gamma carboxyglutamate protein; osteocalcin
BMC	bone mineral content
BMD	bone mineral density
CKD	chronic kidney disease
CKD-MBD	chronic kidney disease–mineral bone disorders
CON	control group
dp-ucMGP	desphospho-uncarboxylated matrix Gla protein
FOXO1	forkhead box transcription factor 1
GGCX	γ-glutamyl carboxylase
Gla	gamma carboxyglutamic acid
Gla-OC	carboxylated osteocalcin
Glu	glutamic acid
Glu-OC	undercarboxylated osteocalcin
HD	hemodialysis
MGP	matrix gla protein
MKs	menaquinones
MK4	menaquinone 4
MK7	menaquinone 7
OC	osteocalcin
PTH	parathyroid hormone
R1	metaphyseal area of femur
R2	diaphyseal area of femur
RUNX2	Runt-related transcription factor 2
UBIAD1	UbiA prenyltransferase domain-containing 1 protein
ucMGP	uncarboxylated matrix gla protein
ucOC	uncarboxylated osteocalcin
VK	vitamin K
VK1	vitamin K1; phylloquinone
VK2	vitamin K2; menaquinones
VKDPs	vitamin K-dependent proteins
VKORC1	vitamin K epoxide reductase complex subunit 1
